# Increased RNA Transcription of Energy Source Transporters in Circulating White Blood Cells of Aged Mice

**DOI:** 10.3389/fnagi.2022.759159

**Published:** 2022-02-03

**Authors:** Yukiko Takeuchi, Orie Saino, Yuka Okinaka, Yuko Ogawa, Rie Akamatsu, Akie Kikuchi-Taura, Yosky Kataoka, Mitsuyo Maeda, Sheraz Gul, Carsten Claussen, Johannes Boltze, Akihiko Taguchi

**Affiliations:** ^1^Department of Regenerative Medicine Research, Foundation for Biomedical Research and Innovation at Kobe, Hyogo, Japan; ^2^Multi-Modal Microstructure Analysis Unit, RIKEN-JEOL Collaboration Center, RIKEN, Hyogo, Japan; ^3^Laboratory for Cellular Function Imaging, RIKEN Center for Biosystems Dynamics Research, RIKEN, Hyogo, Japan; ^4^Fraunhofer Institute for Translational Medicine and Pharmacology ITMP, Hamburg, Germany; ^5^Fraunhofer Cluster of Excellence for Immune-Mediated Diseases (CIMD), Hamburg, Germany; ^6^School of Life Sciences, University of Warwick, Coventry, United Kingdom

**Keywords:** aging, white blood cell, RNA transcription, energy source transporter, metabolism related gene, neurogenesis, hippocampus

## Abstract

Circulating white blood cells (WBC) contribute toward maintenance of cerebral metabolism and brain function. Recently, we showed that during aging, transcription of metabolism related genes, including energy source transports, in the brain significantly decreased at the hippocampus resulting in impaired neurological functions. In this article, we investigated the changes in RNA transcription of metabolism related genes (glucose transporter 1 [Glut1], Glut3, monocarboxylate transporter 4 [MCT4], hypoxia inducible factor 1-α [Hif1-α], prolyl hydroxylase 3 [PHD3] and pyruvate dehydrogenase kinase 1 [PDK1]) in circulating WBC and correlated these with brain function in mice. Contrary to our expectations, most of these metabolism related genes in circulating WBC significantly increased in aged mice, and correlation between their increased RNA transcription and impaired neurological functions was observed. Bone marrow mononuclear transplantation into aged mice decreased metabolism related genes in WBC with accelerated neurogenesis in the hippocampus. *In vitro* analysis revealed that cell-cell interaction between WBC and endothelial cells via gap junction is impaired with aging, and blockade of the interaction increased their transcription in WBC. Our findings indicate that gross analysis of RNA transcription of metabolism related genes in circulating WBC has the potential to provide significant information relating to impaired cell-cell interaction between WBC and endothelial cells of aged mice. Additionally, this can serve as a tool to evaluate the change of the cell-cell interaction caused by various treatments or diseases.

## Introduction

Circulating white blood cells (WBC) have been shown to contribute to the maintenance of cerebral circulation, metabolism, development, neurogenesis and brain function (Taguchi et al., 2004a,^2008^; Filiano et al., 2017; Smith et al., 2018; Pasciuto et al., 2020; Runtsch et al., 2020). Intravenous administration of immature blood cells, such as cord blood derived CD34-positive cells or bone marrow mononuclear cells (BM-MNC), have been shown to improve cerebral circulation and functions after ischemia (Taguchi et al., 2004b; Nakano-Doi et al., 2010). However, the direct link between circulating or intravenously injected cells, and cerebral circulation, metabolism, and function has to date been unclear.

Gap junction is an intercellular channel that allows direct diffusion of ions and small molecules, including metabolic substances, between adjacent cells. One gap junction channel is composed of two hemichannels termed connexons, each one being assembled of six proteins called Connexins (Okamoto et al., 2021). Connexins are a large family of proteins, and Connexin 43 (Cx43) is the most widespread Connexin in the cardiovascular system and is involved both in normal physiological and pathological conditions (Yuan et al., 2015). In the vascular system, connections between endothelial, pericyte and smooth muscle cells via gap junction, as well as between endothelial cells, allow for signaling to take place to endothelial cells as well as other related components (Nielsen et al., 2012). Recently, we showed that intravenously injected BM-MNC supplies an energy source, such as glucose, to endothelial cells via gap junction and activates injured endothelial cells by induction of hypoxia inducible factor 1-α (Hif1-α), which is one of the major activators of metabolism related genes (Kim et al., 2006), in a cerebral ischemia model (Kikuchi-Taura et al., 2020). Intravenous BM-MNC transplantation in aged mice was shown to increase transcription of glucose transporter 1 (Glut1) at hippocampus followed by improved memory functions through gap junction mediated cell-cell interaction between transplanted BM-MNC and endothelial cells (Takeuchi et al., 2020). Furthermore, we have shown that intravenously transplanted mesenchymal stem cells remove the energy source from endothelial cells via gap junction, and suppress their excessive activation in a cerebral ischemia model (Kikuchi-Taura et al., 2021). These findings indicate that circulating cells have a significant effect on the regulation of cerebral circulation, metabolism, and function via gap junction. These findings are consistent with our previous findings that patients with decreased number of circulating hematopoietic stem cells, which express Connexin (Pfenniger et al., 2013) and contain significant amount of glycolysis substrates due to anaerobic metabolism (Takubo et al., 2013), showed impaired cerebral circulation and function (Taguchi et al., 2004a,^2008^).

Cerebral metabolism and function generally deteriorate during aging (Cunnane et al., 2020). It has been shown that metabolites pass through the gap junction (Okamoto et al., 2021) and expression of gap junctions are increased in early postnatal development of central nerve system and after brain injury (Belousov and Fontes, 2014). However, the significance of metabolite transfer via gap junction in the brain is not fully elucidated. Taken together, we hypothesize that impaired cerebral metabolism and function observed in aged animals would correlate to the impaired metabolism of circulating WBC. In this article, we had expected decreased expression of metabolism related genes in WBC with impaired brain function during aging, but found their expression increased with aging.

## Materials and Methods

All animal experiments were approved by the Animal Care and Use Committee of Foundation for Biomedical Research and Innovation at Kobe and comply with the Guide for the Care and Use of Animals published by the Japanese Ministry of Education, Culture, Sports, Science and Technology. Experiments and results are reported according to the ARRIVE guidelines.

### Quantitative PCR (qPCR) Analysis of Circulating White Blood Cells, Hippocampus, and Human Umbilical Vein Endothelial Cells

Male CB-17 mice (C.B-17/Icr- + / + Jcl, Oriental Yeast, Tokyo, Japan) aged 5, 10, 52, and 104 weeks were used for this study (*N* = 5, each). Minimal variation of cerebrovascular structure is known in CB-17 mice (Taguchi et al., 2010) and CB-17 mice were used for the experiment of cerebrovascular disorders (Kasahara et al., 2013; Takeuchi et al., 2020). After deep anesthesia by pentobarbital, a blood sample was obtained by the puncture to left ventricle of heart. Total RNA was isolated using RNeasy Plus Universal Mini Kit (Qiagen, CA, United States) according to the manufacturer’s instructions. cDNA was synthesized from 1μg total RNA using PrimeScript™ II 1st strand cDNA Synthesis Kit (TAKARA, Kyoto, Japan). Transcription of mRNA was analyzed using PowerUp™ SYBR™ Green Master Mix (Applied Biosystems, CA, United States) and the Agilent AriaMx real time PCR System. 18S ribosomal RNA was used for the reference gene. Relative quantification of RNA was analyzed by phaffle method. For the comparison of RNA transcription of Connexin 43 (Cx43) between young and aged mice, 5 and 80 weeks old Male CB-17 mice were used (*N* = 6, each). The list of target genes, primer sequences, and amplification protocols of qPCR are shown in Table 1.

**TABLE 1:** Target genes, primer list and amplification protocol of qPCR.

Gene	NCBI accession no.			Sequences
18S	NR_003278.3	Forward		ACTCAACACGGGAAACCTCACC
		Reverse		CCAGACAAATCGCTCCACCA
Mouse Glut1	NM_011400.3	Forward		TGGCGGGAGACGCATAGTTA
		Reverse		CTCCCACAGCCAACATGAGG
Mouse Glut3	NM_011401.4	Forward		GAGGAACACTTGCTGCCGAG
		Reverse		CTGGAAAGAGCCGATCGTGG
Mouse MCT4	NM_001038653.1	Forward		GGCTGGCGGTAACAGAGTA
		Reverse		CGGCCTCGGACCTGAGTATT
Mouse Hif1-α	NM_001313919.1	Forward		AGCCAGCAAGTCCTTCTGAT
		Reverse		AGGCTGGGAAAAGTTAGGAGTG
Mouse PHD3	NM_028133.2	Forward		ATCCACATGAAGTCCAGCCC
		Reverse		ACACCACAGTCAGTCTTTAGCA
Mouse PDK1	NM_172665.5	Forward		TGCAAAGTTGGTATATCCAAAGCC
		Reverse		TGTGCCGGTTTCTGATCCTT
Mouse Cx37	NM_008120.3	Forward		CAGCTGCGCGCTATTTAAGG
		Reverse		CCATGTTTCCAGGGCCTCTC
Mouse Cx43	NM_010288.3	Forward		GAGTTCCACCACTTTGGCGT
		Reverse		GTGGAGTAGGCTTGGACCTT
Human Cx37	NM_002060.2	Forward		CACCATGCCCCACCTACAAT
		Reverse		TGGGGGTTTTTGGCCATTC
Human Cx43	NM_000165.4	Forward		AGGAGTTCAATCACTTGGCGT
		Reverse		CCCTCCAGCAGTTGAGTAGG

**Segment**	**Plateau**	**Temperature (°C)**	**Duration time (seconds)**	**Cycle (times)**

Hot Start	1	50	180	1
Hot Start 2	1	95	180	1
Amplification	1	95	5	40
Amplification	2	60	30	40
Melt	1	95	30	1
Melt	2	65	30	1
Melt	3	95	30	1

Brain tissue was harvested from young (5W) and aged (more than 80 weeks) mice (*N* = 7, each). After harvesting the brain, coronal sections (4 mm thick) of the forebrain between 2 mm and 6 mm from frontal pole were cut followed by immersion in RNAlater (Thermo Fisher, Waltham, MA, United States) to prevent RNA degradation. Under stereoscopic microscope (Olympus, Tokyo, Japan), the part of the hippocampus, was dissected by medical tweezers, as we described previously (Takeuchi et al., 2020). Total RNA was isolated and cDNA was synthesized from 1 μg total RNA. The list of target genes, primer sequences, and amplification protocols are shown in Table 1.

Human umbilical vein endothelial cells (HUVEC, Kurabo, Osaka, Japan) were cultured with medium, serum and growth factors (HuMedia-EB2, Kurabo). Total RNA of HUVEC (1 × 10^5^ cells) at passage 3 and 10 were was isolated and expression of Cx43 and Connexin 37 (Cx37) were evaluated by qPCR (*N* = 4). The list of target genes, primer sequences, and amplification protocols of qPCR are shown in Table 1.

### Transfer of Low Molecular Weight Fluorescence Molecules in Cytoplasm of White Blood Cells to Endothelial Cells *in vitro*

Male CB-17 mice aged 5 and 80 weeks old were used for this study (*N* = 5, each). Blood sample was obtained by a puncture to the left ventricle of the heart and red blood cells were removed using lysing buffer (BD Biosciences, Woburn, MA, United States) (Muirhead et al., 1986). WBC were incubated with 5 μM of BCECF-AM (2′,7′-bis-(2-carboxyethyl)-5-(and-6)-carboxyfluorescein, acetoxymethyl ester, Dojindo, Kumamoto Japan) for 30 min at 37°C as we described previously (Kikuchi-Taura et al., 2020). BCECF-AM was converted to BCECF (2′,7′-bis-(2-carboxyethyl)-5-(6)-carboxyfluorescein) in the cytoplasm and BCECF loaded WBC were washed twice with PBS before use. HUVEC in passage 6 were used in the subsequent experimental work. BCECF loaded WBC (1 × 10^6^ cells in 20 μl PBS) and HUVEC (1 × 10^5^ cells in 100 μl PBS) were co-cultured at 37°C for 3 h. After co-incubation, the cell mixture was washed twice with PBS, and stained with PE-conjugated anti-human CD31 antibody (BD Biosciences, Woburn, MA, United States), FITC-conjugated anti-mouse CD45 antibody (BD Biosciences, Woburn, MA, United States) and 7-AAD (7-Amino-Actinomycin D) (BD Biosciences, Woburn, MA, United States). The level of BCECF in HUVEC (CD31-positive, CD45-negative and 7AAD-negative) was evaluated using a FACS Calibur fluorescence activated cell sorter (BD Biosciences, Woburn, MA, United States). Cell populations that communicate with HUVEC via gap junction were characterized using FACS as described previously (Kikuchi-Taura et al., 2021).

### Effect of Gap-Junction Mediated Cell-Cell Interaction on Gene Expression of White Blood Cells by Co-culture With Endothelial Cells

HUVEC were treated with gap junction blocker (0.01 v/v 1-octanol. Wako, Osaka, Japan) or PBS for 10 min at 37°C. A blood sample was obtained by a puncture to the left ventricle of the heart of male CB-17 mice (5 weeks old) and co-cultured at 37°C for 3 h with HUVEC that were treated or non-treated by gap junction blocker. The change of RNA transcription was evaluated by qPCR with mouse specific primers.

### BM-MNC Transplantation to Aged Mice

Bone marrow was obtained from 5-week-old syngeneic male CB-17 mice and BM-MNC were prepared by Ficoll-Paque (GE-Healthcare, Little Chalfont, United Kingdom) density-gradient centrifugation as described elsewhere (Kikuchi-Taura et al., 2020). Aged (more than 80 weeks) mice received 1 × 10^5^ BM-MNC in 100 μl PBS or PBS alone through the tail vein (5 times in total), and young (5 weeks) mice received PBS (*N* = 5, each). Each injection was performed without anesthesia and injection was finished in 10 s. Mouse behavior was evaluated using wire hang test and passive avoidance test before blood sample collection. The experimental design is shown in Supplementary Figure 1A. The Passive avoidance test is a fear-aggravated test used to evaluate learning and memory in rodent models. The apparatus (TMS-2; Melquest, Toyama, Japan) was divided into two compartments, an illuminated compartment (120 mm × 120 mm × 135 mm) and a dark compartment (120 mm × 120 mm × 135 mm), that were connected via a guillotine door. The dark compartment was equipped with a grid floor (6 mm in diameter spaced at 10 mm) through which a footshock (0.2 mA, 3 s) could be delivered. In the conditioning and acquisition phases, each mouse was placed in an illuminated compartment and habituated for 10 s before door opening. In the conditioning phase, a mild electric foot shock was administered 10 s after each mouse crossed the adjacent dark compartment and door closing. On the next day, each mouse was again placed in the illuminated compartment and habituated for 10 s, and the time to cross over to the dark compartment after door opening, up to a maximum of 180 s, was recorded as the test phase (Ogawa et al., 2020). The wire hang test evaluates muscular strength or motor function. In this test, each mouse was placed on the center of wire mesh plate (450 mm × 300 mm; mesh wire was 1.3 mm in diameter and spaced at 12 mm intervals, KK23-8331; Kohnan, Osaka, Japan) and allowed to accommodate to this environment for 5 s. The wire mesh plate was inverted and secured to the top of a cubic transparent open-topped glass box (250 mm × 250 mm × 250 mm). Latency to fall was recorded, up to a maximum of 180 s. This trial was repeated five times with an interval of 1 min and the average time to fall was calculated (Ogawa et al., 2020).

For immune historical analysis, aged mice (more than 80 weeks) received 1 × 10^5^ BM-MNC in 100 μl PBS or PBS alone intravenously totally five times every 2 days from tail vein, and young (5 weeks) mice received PBS (*N* = 6,7,8, for aged + PBS, aged + BM-MNC and young + PBS, respectively). Twenty-four hours after the last injection, the brain was removed, fixed with 2% paraformaldehyde (PFA; Thermo Fisher, Waltham, MA, United States), and cut into coronal sections (20 μm) using a vibratome (Leica, Wetzlar, Germany). Sections were immunostained with primary antibodies against Nestin (Merck Millipore,1:200), Doublecortin (DCX; Merck Millipore, 1:350) and DAPI (Thermo Fisher, 1:1,000). Confocal images were obtained with fluorescence microscope (BZ-X810: Keyence, Osaka Japan). The number of Nestin positive fibers and DCX positive cells at the granular cell layer and sub granular zone of dentate gyrus, respectively, in the hippocampus of aged mice were counted by blinded investigator. The number of positive cells per 1 mm of each zone was evaluated.

### Increased Expression of Gap-Junction in HUVEC by Co-culture With BM-MNC

HUVEC (1 × 10^5^ cells in 100 μl PBS) and BM-MNC (1 × 10^6^ cells in 20 μl PBS) were co-cultured at 37°C for 3 h, and the change in expression of Cx43 in HUVEC was evaluated with PE-conjugated anti-human CD31 antibody, FITC-conjugated anti-human Cx43 antibody (Santa Cruz Biotechnology, Santa Cruz, CA, United States) and 7-AAD (7-Amino-Actinomycin D) by FACS Calibur fluorescent cell sorter.

### Statistics

Statistical comparisons among groups were performed using one-way ANOVA (analysis of variance) followed by *post hoc* analysis using Steel-Dwass test or Dunnett’s test. Correlation between RNA transcription of metabolism related genes in circulating WBC and neurological functions was evaluated with linear regression analysis. Individual comparisons were performed using Student’s *t*-test. All data are shown as mean ± SD.

## Results

### Change in RNA Transcription of Metabolism-Related Genes in Circulating Whites Blood Cells With Aging

**FIGURE 1:**
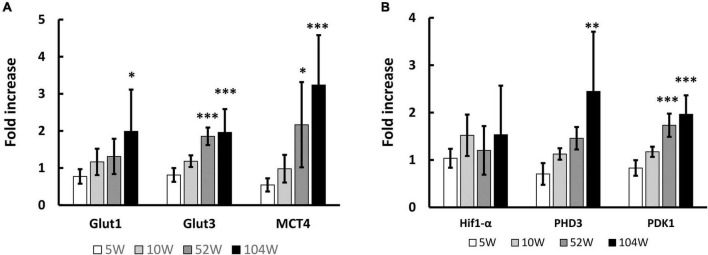
Change in RNA transcription in circulating WBC with aging. **(A)** Significant increase of RNA transcription of energy source transport genes, including Glut1, Glut3 and MCT4, in WBC was observed with aging. **(B)** Similarly, RNA transcriptions of Hif1-α down-stream genes, PHD3 and PDK1, in WBC significantly increased with aging. *p < 0.05, ^**^p < 0.01, ^***^p < 0.001 by ANOVA followed by *post-hoc* analysis using Dunnett test (versus 5W). *N* = 5 in 10W, 104W and *N* = 6 in 5W, 52W **(A,B)**.

We previously observed that transcription of metabolism related genes, including energy source transport genes, in the brain are significantly decreased with aging with impaired neurological functions. We expected similar changes in WBC and investigated the sequential change in RNA transcription of these genes in circulating WBC. Contrary to our expectation, the energy source transport genes, including Glut1, Glut3, and monocarboxylate transporter 4 (MCT4), in WBC significantly increased with aging (Figure 1A). Hif1-α is one of the major transcriptional factors that regulates the expression of energy source transporters (Masoud and Li, 2015; Zorzano et al., 2005) and has been shown to decrease with aging in the brain (Takeuchi et al., 2020). The transcription of Hif1-α and its down-stream genes, prolyl hydroxylase 3 (PHD3) and pyruvate dehydrogenase kinase 1 (PDK1), in circulating WBC were investigated and it was found that transcription of PHD3 and PDK1 increased with aging (Figure 1B).

### Cell-Cell Interaction Between WBC and Endothelial Cells via Gap Junction

**FIGURE 2:**
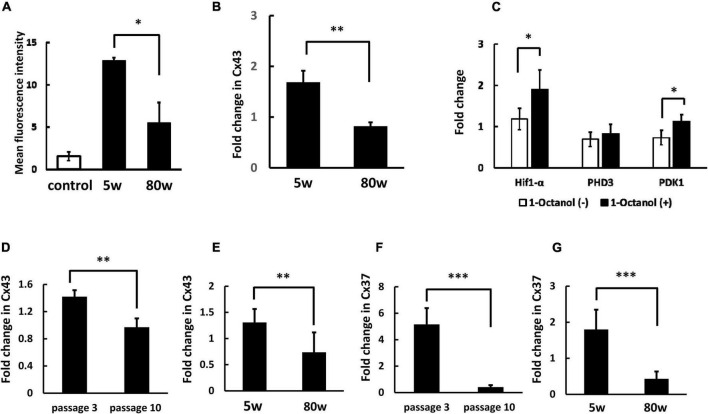
Decreased cell-cell interaction between endothelial cells and WBC of aged mice. **(A)** Transfer of low molecular weight molecule (BCECF) from WBC to HUVEC significantly decreased at WBC of aged mice, compared with young mice. Control indicates background fluorescence level HUVEC without co-incubation of WBC. **(B)** The RNA transcription of Cx43 in WBC was significantly decreased with aging. **(C)** Blockade of cell-cell interaction between WBC and HUVEC via gap junction increased the expression of Hif1-α and PDK1 in WBC. **(D)** The RNA transcription of Cx43 in HUVEC was significantly decreased with passaging. **(E)** The RNA transcription of Cx43 at hippocampus was significantly decreased with aging. **(F)** The RNA transcription of Cx37 in HUVEC was significantly decreased with passaging. **(G)** The RNA transcription of Cx37 at hippocampus was significantly decreased with aging. *p < 0.05 versus 5W (*N* = 5 in group **A**), ^**^p < 0.01 versus 5W (*N* = 6 in group **B**) by Student’s *t*-test. *p < 0.05 versus 1-Octanol (–) (*N* = 4 in group **C**). ^**^p < 0.01, ^***^p < 0.001 versus passage 3 (*N* = 4 in group **D, F**) by Student’s *t*-test. ^**^ *p* <0.01, ^***^p < 0.001 versus 5W (*N* = 7 in group **E, G**) by Student’s *t*-test.

We have shown low molecular weight molecules are transferred from BM-MNC to endothelial cells via gap junction mediated direct cell-cell interaction (Kikuchi-Taura et al., 2020; Takeuchi et al., 2020). To investigate gap junction mediated cell-cell interaction between WBC and endothelial cells, low molecular weight fluorescence compound, namely BCECF, was loaded to WBC and co-cultured with HUVEC. Similar to BM-MNC, transfer of BCECF from WBC to HUVEC was observed and the level of BCECF transfer significantly decreased in WBC of aged mice, relative to that of young mice (Figure 2A). The change of RNA transcriptions with aging was evaluated and found the level of Cx43 in WBC was significantly decreased in aged mice, compared with young mice (Figure 2B). To confirm the significance of gap junction mediated cell-cell interaction on RNA transcription in WBC, WBC were co-cultured with HUVEC with or without blockade of gap junction. A significant increase of RNA transcription of Hif1-α and PDK1 was observed following blockade of gap junction (Figure 2C). These findings show the importance of gap junction mediated cell-cell interaction in the decrease of metabolism related gene expression in WBC, mainly monocytes and lymphocytes. The change in RNA transcription with passage number was evaluated in HUVEC and it was found that the level of Cx43 significantly decreased during passaging (Figure 2D). The change in RNA transcription with aging at hippocampus was evaluated and it was found that the level of Cx43 significantly decreased in aged mice, compared with young mice (Figure 2E). The change of RNA transcription of Cx37 was evaluated and found its expression decreased in HUVEC during passaging (Figure 2F) and in hippocampus with aging (Figure 2G).

### Correlation in RNA Transcription of Metabolism Related Genes in Circulating White Blood Cells and Neurological Function in Aging Mice

**FIGURE 3:**
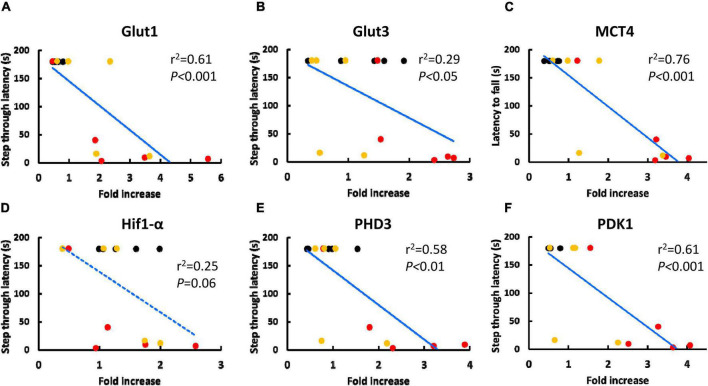
Correlation between the score of passive avoidance test and RNA transcription of metabolism related genes in circulating WBC. **(A-F)** Correlation between increased in RNA transcription of Glut1 **(A)**, Glut3 **(B)**, MCT4 **(C)**, Hif1-α **(D)**, PHD3 **(E)** and PDK1 **(F)** and shorting of step through latency in passive avoidance test. Black, red or yellow dots indicate young mice, aged mice with PBS injection or aged mice with BM-MNC transplantation, respectively. Blue solid or dashed line indicate statistically highly (*p* < 0.05 and | r| > 0.7) or moderately (*p* < 0.05 and | r| > 0.4) correlating, respectively, by linear regression analysis (*N* = 15).

Next, we evaluated the correlation between RNA transcription of metabolism related genes in circulating WBC and neurological function in mice, including aged mice that received BM-MNC transplantation. Figures 3A-F shows the correlation between increased RNA transcription of metabolism related genes and shortening of step through latency in passive avoidance test. A strong and significant correlation (| r| > 0.7 and *p* < 0.05) was observed for Glut1, MCT4, PHD3, and PDK1. Supplementary Figure 2 shows the correlation between increased RNA transcription of metabolism related genes and shortening of latency to fall in wire hang test. A strong and significant correlation (| r| > 0.7 and *p* < 0.05) was observed for MCT4, PHD3, and PDK1. Supplementary Figures 3, 4 show the correlation in aged mice, including those that received PBS or BM-MNC but not young mice, in the passive avoidance test (Supplementary Figure 3) and in the wire hang test (Supplementary Figure 4). A strong and significant correlation was observed for RNA transcription of MCT4 and PHD3. It should be noted that, a statistically significant reduction of the latency in passive avoidance test and wire hang test in aged mice with PBS, compared with young mice, was reported in our previous report. In contrast, BM-MNC injection to aged mice improved the scores of both tests and there had been no significant difference in the latencies between in aged mice with BM-MNC and young mice, though no statistically significant difference had been observed between in the scores of aged mice that received PBS or BM-MNC (Takeuchi et al., 2020).

**FIGURE 4:**
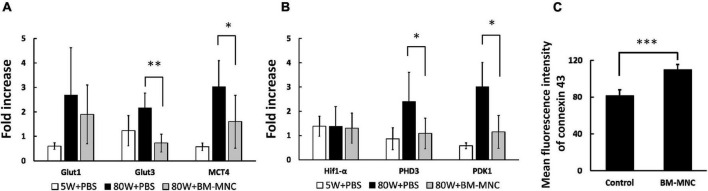
BM-MNC transplantation decreased RNA expression of metabolism related genes in WBC. **(A,B)** BM-MNC transplantation to aged mice decreased the expression of Glut3, MCT4 **(A)**, PHD3 and PDK1 **(B)** in circulating WBC. **(C)** Co-culture with BM-MNC significantly increased the expression of Cx43 in endothelial cells. *p < 0.05 and ^**^p < 0.01 vs. 80W + PBS by ANOVA followed by *post hoc* analysis using Dunnett’s test (control, 80W + PBS; *p* values between aged mice were displayed in the figure). *N* = 5 in each group **(A,B)**. ^***^p < 0.001 by Student’s *t*-test. *N* = 6 in each group **(C)**.

Mice that received BM-MNC transplantation showed decreased RNA expression of Glut3, MCT4, PHD3, and PDK1 in circulating WBC (Figures 4A,B). To investigate the possible cause of decreased level of these genes by BM-MNC transplantation, endothelial cells were co-cultured with BM-MNC. We observed increased expression of Cx43 in HUVEC when co-cultured with BM-MNC (Figure 4C). These findings indicated that BM-MNC transplantation has a potential to improve cell-cell interaction between circulating WBC and endothelial cells through enhancing expression of Connexin on endothelial cells.

**FIGURE 5:**
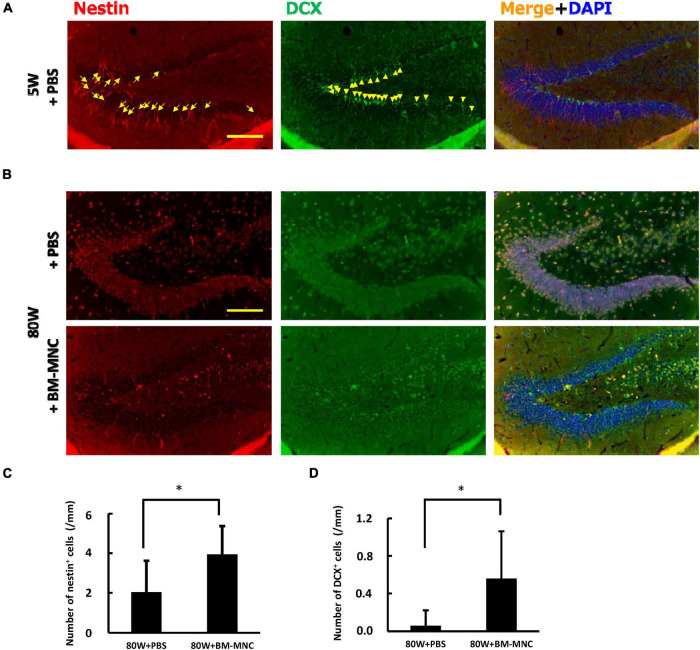
BM-MNC transplantation activates age-related impaired neurogenesis at hippocampus. **(A)** A large number of Nestin-positive (arrow) and/or DCX-positive (allow head) neuronal progenitor cells were observed at dentate gyrus in young mice. **(B)** In contrast, only few Nestin-positive and/or DCX-positive neuronal progenitor cells were observed in aged mice that receive PBS or BM-MNC. **(C,D)** Quantitative analysis revealed that BM-MNC transplantation to aged mice increased the number of Nestin-positive **(C)** and DCX-positive neuronal progenitor cells **(D)**. *N* = 6 and 7 in PBS and BM-MNC transplanted group, respectively **(C,D)**. *p < 0.05 by Student’s *t*-test. Scale bar: 100 μm **(A,B)**.

To further investigate the link between BM-MNC transplantation and increased memory function, the level of neurogenesis at hippocampus was evaluated using anti-Nestin and anti-DCX antibody, and it was found that the level of neurogenesis significantly increases with BM-MNC transplantation in aged mice (Figures 5A-D). These findings are consistent with our previous report that BM-MNC transplantation ameliorates memory disorder in aged mice (Takeuchi et al., 2020).

## Discussion

In this article, we have demonstrated that RNA transcription of metabolism related genes in circulating WBC, including Glut1, Glut3, MCT4, PHD3, and PDK1, are significantly increased during aging. Our results indicated that this is, at least in part, due to the decrease of cell-cell interactions to endothelial cells. Our findings indicate that the level of RNA transcription of metabolism related genes in circulating WBC can be potentially utilized as markers for decreased cell-cell interaction between circulating WBC and endothelial cells during aging.

**FIGURE 6:**
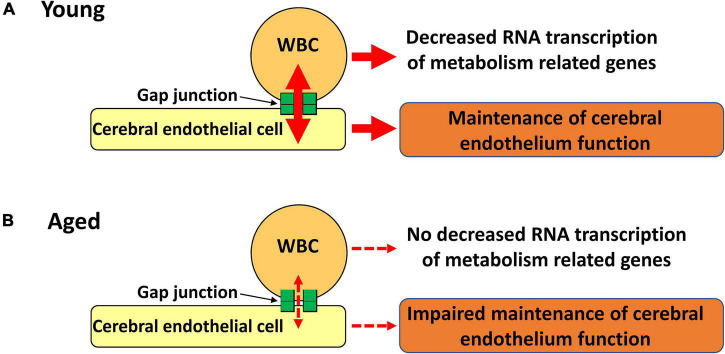
Schematic illustration of our hypothesis. **(A)** Gap junction mediated cell-cell interaction between circulating WBC and endothelial cells decrease RNA transcription of metabolic related genes in WBC with maintenance of endothelial cell function. **(B)** Impaired gap junction mediated cell-cell interaction results in no decreased RNA transcription of metabolic related genes in WBC with impaired maintenance of endothelial cell function.

Importantly, circulating WBC are known to contribute to the development and maintenance of brain function (Taguchi et al., 2004a,^2008^; Filiano et al., 2017; Smith et al., 2018; Pasciuto et al., 2020; Runtsch et al., 2020). Recently, we demonstrated that direct cell-cell interaction between intravenously transplanted BM-MNC and endothelial cell via gap junction activates Hif1-α at endothelial cells followed by improvement of brain function (Kikuchi-Taura et al., 2020). In this article, we demonstrate that cell-cell interaction between circulating WBC and endothelial cell decreases with aging with impaired neurological function. As endothelial, pericyte, and smooth muscle cells are connected via gap junction in cerebral vasculature (Zhao et al., 2018), it would not be so surprising that metabolites and/or signals transfer between circulating cells and cerebral endothelial cells have a significant impact on cerebral circulation and function. Recently, we have demonstrated that intravenously transplanted mesenchymal stem cells remove energy source from endothelial cells via gap junction, and suppress excessed activation of endothelial cells in cerebral ischemia model (Kikuchi-Taura et al., 2021). Although further studies are required to fully elucidate the flow of metabolites and signals via gap junction with brain responses, our findings provide a novel paradigm linking cells in systemic circulation, endothelial cells and cerebral function (Figure 6).

Hif1-α is one of the major transcriptional factors that regulates the expression of energy source transporters (Zorzano et al., 2005; Masoud and Li, 2015) and PHD3 and PDK1 are known to be down-stream genes of Hif1-α(Ognibene et al., 2017; Takeuchi et al., 2020). As the activity of HIF1α protein can be regulated by its disassembly by HIF-prolyl hydroxylase in an oxidant dependent manner (Hill et al., 1992), our results shown in Figures 1B, 4B, show there was a discrepancy between Hif1-α and PHD3 expression, which would be explained by the change of degradation rate rather than RNA transcription. Our previous results showed movement of small molecules, including glucose, from intravenously transplanted BM-MNC activates Hif1-α in recipient endothelial cells via gap junction mediated cell-cell interaction (Kikuchi-Taura et al., 2020). In this article, we show that movement of small molecules to endothelial cells via gap junction inactivates Hif1-α in donor WBC. These findings show that small molecule movement mediated Hif1-α regulation is the key mechanism for changes of metabolism related gene expression at circulating WBC and endothelial cells.

In this article, we have demonstrated that BM-MNC transplantation into aged mice enhanced neurogenesis in hippocampus and decreased metabolic related genes in WBC. These findings are consistent with our previous report that BM-MNC transplantation to aged mice increases Glut1 and Na^+^/K^+^ ATPase expression in the hippocampus and ameliorates memory disorder (Takeuchi et al., 2020). New neurons in the hippocampus are known to have a pivotal role in improving memory (Moreno-Jimenez et al., 2019; Tobin et al., 2019) and our findings will have a significant impact that links cell therapy, improved neurogenesis, and improvement of memory. Once the mechanism of metabolite transfer via gap junction and activation of Hif1-α in brain is understood, this offers the potential to improve and develop novel therapies for dementia in the elderly whose main symptom is the disability of memory.

*In vitro* analysis revealed that BM-MNC enhanced the expression of Cx43 in endothelial cells. The turnover of Connexin molecules is only a few hours (Musil et al., 2000) that would require a significant amount of energy source. Although the reason of the fast turnover of Connexin and the regulator of Connexin transcription are unclear (Laird, 2006; Nielsen et al., 2012), it would not be surprising that gap junction mediated activation of Hif1-α increases energy source uptake and that upregulates the expression of Connexin. The enhanced expression of Cx43 in endothelial cells by BM-MNC transplantation would explain our result that aged mice that received BM-MNC transplantation showed decreased expression of metabolism related genes in circulating WBC. Our findings also show the potential that quantification of these metabolism related genes in WBC can serve as a tool to evaluate the change of the cell-cell interaction between WBC and endothelial cells caused by treatments that may affect the cell-cell interaction.

Limitations of our study include deciphering the mechanism of cell-cell interaction between WBC and endothelial cells which regulate Hif1-α gene expression in WBC and the differences in gene expression profiles between cell types, such as granulocytes, lymphocytes, and monocytes. Previous findings have shown that the lymphocytes and monocytes express Connexins, although no expression was observed in granulocytes under physiological conditions (Pfenniger et al., 2013). Although further mechanistic studies are required in order to determine the change of gene expression in each cell type, our findings demonstrate that the simple gross analysis of circulating WBC provides a marker for decreased cell-cell interaction between circulating WBC and endothelial cells with aging in mice. This parameter in mice does not require cell separation or cell specific group analysis and therefore is beneficial for screening individuals to assess the cell-cell interaction between circulating WBC and endothelial cells in mice. This information would also provide novel insights into pathology of various cardiovascular diseases. Another limitation is that we have used one strain of mice. To generalize the results obtained in this experiment, further studies using other strains and animal are required, especially for the assessment of human WBC with aging.

In conclusion, our novel findings show the potential that the simple gross analysis of RNA transcription of metabolism related genes in circulating WBC can provide a highly relevant insight into impaired cell-cell interaction between WBC and endothelial cells. This can also serve as a tool to evaluate the change of the cell-cell interaction caused by various treatments, diseases, and aging.

## Data Availability Statement

The original contributions presented in the study are included in the article/Supplementary Material, further inquiries can be directed to the corresponding author/s.

## Ethics Statement

The animal study was reviewed and approved by Animal Care and Use Committee of Foundation for Biomedical Research and Innovation at Kobe.

## Author Contributions

YT, YOk, and AK-T performed the experiments and analyzed data. AT and OS prepared this manuscript the final figures and tables. YOg, RA, YK, MM, SG, CC, and JB supervised the project and revised the manuscript critically for important intellectual content. AT planned and designed the study. All authors gave their approval to the manuscripts.

## Conflict of Interest

The authors declare that the research was conducted in the absence of any commercial or financial relationships that could be construed as a potential conflict of interest.

## Publisher’s Note

All claims expressed in this article are solely those of the authors and do not necessarily represent those of their affiliated organizations, or those of the publisher, the editors and the reviewers. Any product that may be evaluated in this article, or claim that may be made by its manufacturer, is not guaranteed or endorsed by the publisher.

## Abbreviations

WBC: white blood cells
BM-MNC: bone marrow mononuclear cells
Hif1-α: hypoxia inducible factor 1-α
Glut1: glucose transporter 1
Glut3: glucose transporter 3
MCT4: monocarboxylate transporter 4
PHD3: prolyl hydroxylase 3
PDK1: pyruvate dehydrogenase kinase 1
qPCR: Quantitative PCR
Cx43: Connexin 43
Cx37: Connexin 37
BCECF: 2′,7′-bis-(2-carboxyethyl)-5-(6)-carboxyfluorescein
DCX: Doublecortin
HUVEC: Human umbilical vein endothelial cells
ANOVA: analysis of variance.
