# Supplementary material for: Increased RNA Transcription of Energy Source Transporters in Circulating White Blood Cells of Aged Mice
Source: Front Aging Neurosci. 2022 Feb 3;14:759159. doi: 10.3389/fnagi.2022.759159 (PMC8850360; doi:10.3389/fnagi.2022.759159)
Supplement: Supplementary file 1 [file Data_Sheet_1.docx]

Supplementary Material

## 1. Supplementary Figures

## 1.1. Supplementary Figure 1


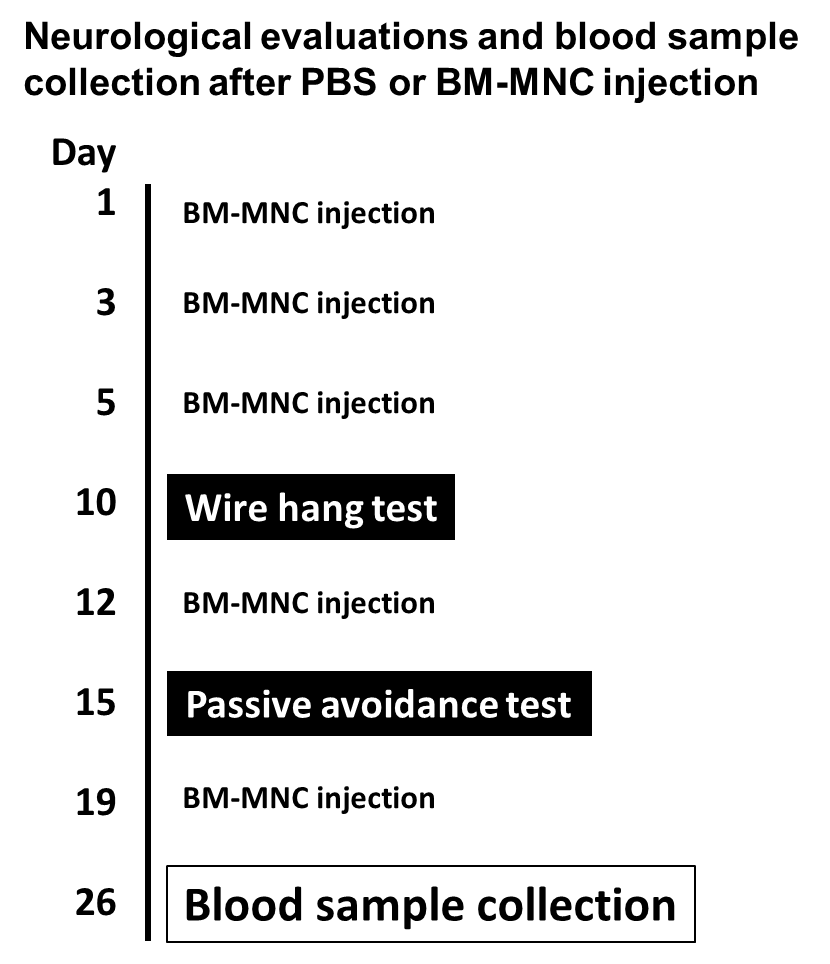


## Supplementary Figure 1. Design of neurological evaluations and blood sample collection after PBS or BM-MNC injection. Schematic illustration of experimental design.

**1.2. Supplementary Figure 2**

**
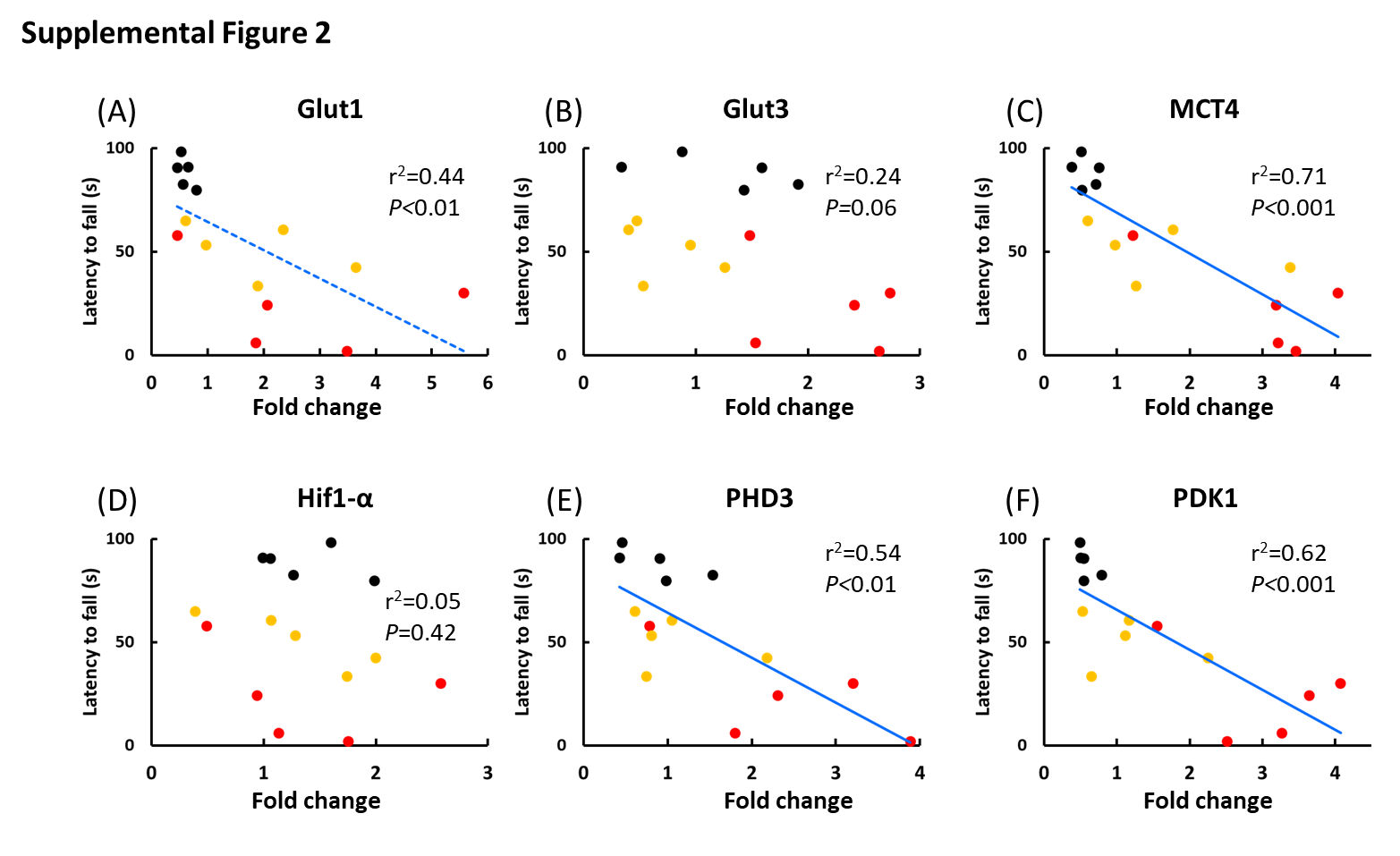
**

**Supplementary Figure 2.** **Correlation between the score of wire hang test and RNA transcription of metabolism related genes in circulating WBC.**  (A-F) Correlation between increased in RNA transcription of Glut1 (A), Glut3 (B), MCT4 (C), Hif1-α (D), PHD3 (E) and PDK1 (F). Black, red or yellow dots indicate young mice with PBS injection, aged mice with PBS injection or aged mice with BM-MNC injection, respectively. Blue solid or dashed lines indicate statistically highly (p<0.05 and |r|> 0.7) or statistically moderately (p<0.05 and |r|> 0.4) correlating, respectively, by linear regression analysis (N=15).

**1.4. Supplementary Figure 3**

**
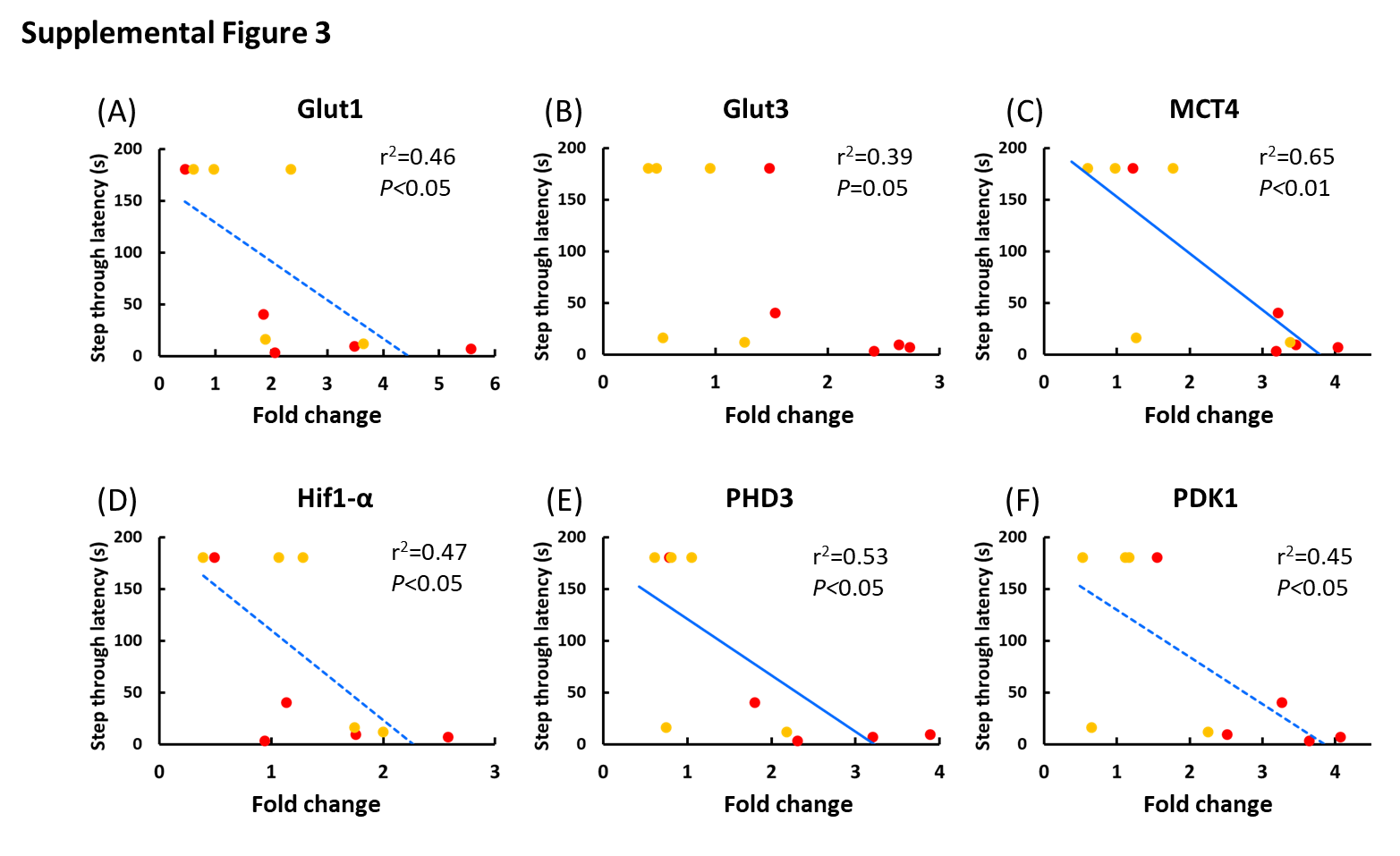
**

**Supplemental Figure 3. Correlation between the score of passive avoidance test and RNA transcription of metabolism related genes in circulating WBC of aged mice.** (A-F) Correlation between increased in RNA transcription of Glut1 (A), Glut3 (B), MCT4 (C), Hif1-α (D), PHD3 (E) and PDK1 (F) and shorting of step through latency in passive avoidance test. Red or yellow dots indicate aged mice with PBS injection or aged mice with BM-MNC injection, respectively. Blue solid or dashed lines indicate statistically highly (p<0.05 and |r|> 0.7) or statistically moderately (p<0.05 and |r|> 0.4) correlating, respectively, by linear regression analysis (N=10).

**1.4. Supplementary Figure 4**


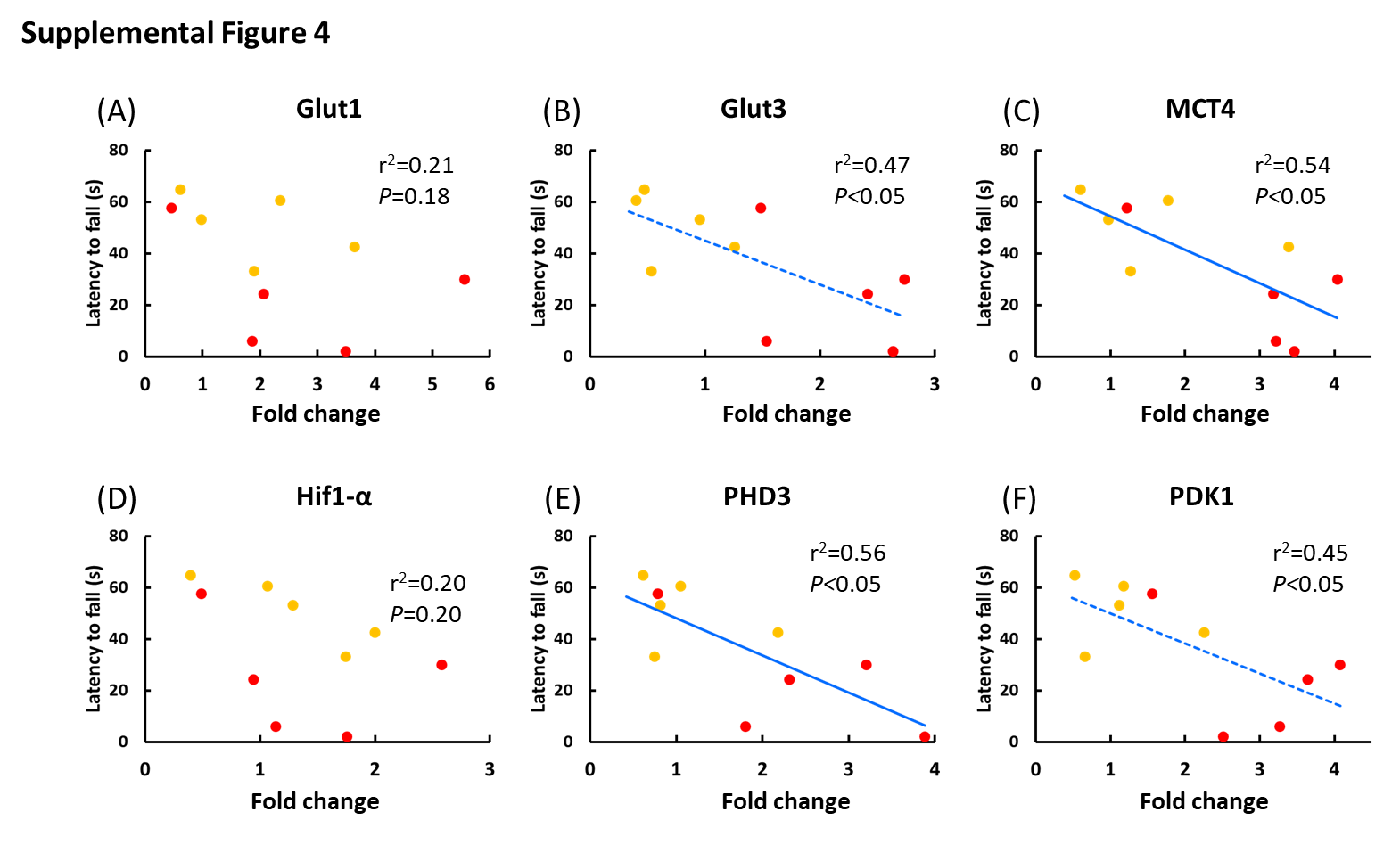


**Supplemental Figure 4. Correlation between the score of wire hang test and RNA transcription of metabolism related genes in circulating WBC of aged mice.** (A-F) Correlation between increased in RNA transcription of Glut1 (A), Glut3 (B), MCT4 (C), Hif1-α (D), PHD3 (E) and PDK1 (F) and shorting latency to fall in wire hang test of aged mice. Red or yellow dots indicate aged mice with PBS injection or aged mice with BM-MNC injection, respectively. Blue solid or dashed line indicate statistically highly (p<0.05 and |r|> 0.7) or statistically moderately (p<0.05 and |r|> 0.4) correlating, respectively, by linear regression analysis (N=10).
